# Risk factors and mortality in patients with pneumonia and elevated troponin levels

**DOI:** 10.1038/s41598-020-78287-1

**Published:** 2020-12-10

**Authors:** Orly Efros, Shelly Soffer, Avshalom Leibowitz, Alexander Fardman, Robert Klempfner, Eshcar Meisel, Ehud Grossman

**Affiliations:** 1 Internal Medicine D, Sheba Medical Center, Tel-Hashomer, Israel; 2 Sackler Faculty of Medicine, Tel-Aviv University, Tel Aviv, Israel; 3 Cardiac Rehabilitation Institute, Leviev Heart Center, Sheba Medical Center, Tel Hashomer, Israel; 4 Internal Medicine Wing, Sheba Medical Center, Tel-Hashomer, Israel

**Keywords:** Diagnostic markers, Predictive markers, Biomarkers, Cardiology, Diseases, Health care, Medical research, Risk factors

## Abstract

Pneumonia in hospitalized patients is associated with myocardial injury. In this study, we evaluated risk factors for myocardial injury in hospitalized patients with pneumonia and its prognostic value. We retrieved all patients who were hospitalized in internal medicine departments in a tertiary medical center between 2008 and 2019 with a diagnosis of pneumonia. From 2008 to 2019 a total of 20,683 adult patients were hospitalized in internal medicine wards in the Sheba Medical Center with a diagnosis of pneumonia, 8195 were tested for troponin levels, and 3207 had elevated levels. Risk factors for elevated troponin levels were age, prior diagnosis of ischemic heart disease, and elevated creatinine level upon admission. The in-hospital mortality and 1-year mortality rate were higher among patients who had elevated troponin levels when using a propensity score-based matched analysis. In conclusion, in hospitalized patients with pneumonia elevated troponin levels have a major impact on prognosis. Hence, troponin levels may be used as another tool of risk stratification for patients hospitalized with pneumonia.

## Introduction

Pneumonia is a common worldwide infectious disease that is associated with a high hospitalization rate and mortality^1,2^. Although pneumonia is an acute condition, the long-term effects of this disease are significant^3^.

Several studies have demonstrated the association between pneumonia and the risk of future cardiac complications. Pneumonia has been shown to predispose to several cardiac conditions including heart failure, cardiac arrhythmia and myocardial infarction (MI)^4,5^. Among patients hospitalized with pneumonia, 2.3–7% were reported with concurrent MI^6,7^. The incidence of MI surges to 15% in patients with severe pneumonia and to 20% in pneumonia patients who experienced clinical failure^7^. The risk for MI among pneumonia patients is most common during the 15 days following the diagnosis of pneumonia, with the highest risk within the first 3 days^8^. The proposed mechanisms underlying the triggers of acute MI in pneumonia include hypoxemia, increased sympathetic activity, increased inflammatory activity within coronary atherosclerotic plaques and endothelial dysfunction^4^.

The in-hospital mortality rate of pneumonia patients with MI is substantial. In a study conducted by Aliberti et al., it was reported that the mortality rate was significantly higher among patients who have had an MI following pneumonia (43%) than among those who developed other cardiovascular events (21%)^6^. Another study demonstrated that the mortality rate remains high even in a follow-up of 2 years^9^.

Endeavors have been made to identify pneumonia patients who are at a greater risk for developing cardiovascular events^10,11^. Several factors have been suggested including old age, previous cardiac history, and severe pneumonia. Further research has pointed to factors specifically linked to higher risk for MI: female sex, liver disease and the presence of severe sepsis^6^.

Previous research focusing on acute MI in pneumonia patients has been limited to a relatively small cohort. The aims of our study were (1) to evaluate the incidence of acute myocardial injury in hospitalized patients with pneumonia, (2) to investigate risk factors for acute cardiovascular events following pneumonia, (3) and to determine the short and long term outcomes for this population. In our research, we used a large database from the biggest tertiary hospital in Israel, comprising of 8195 pneumonia patients who were also tested for troponin I levels.

## Results

### Baseline characteristics

From 2008 to 2019, a total of 20,683 patients over the age of 18 were hospitalized with a diagnosis of pneumonia in the internal medicine wards at the Sheba Medical Center.

Among the 20,683 patients diagnosed with pneumonia, 8195 were tested for troponin levels during their stay, with 3207 (39.1%) of them found to have an elevated level of troponin.

Patients’ characteristics are presented in Tables 1, 2. Patients who were tested for troponin were slightly older than those who were not tested and had more underlying diseases (Table 1). Among patients who were tested for troponin, those who had elevated troponin levels were older and tended to have more underlying diseases than those with normal troponin levels (Table 2). Accordingly, patients with elevated troponin levels were also more likely to be prescribed with angiotensin-converting enzyme inhibitors, beta-blockers, HMG CoA reductase inhibitors (Supplementary 2).

**Table 1:** Characteristics of the patients at baseline.

	Troponin non taken (N = 12,488)	Troponin taken (N = 8195)	P-value
Age, years—median (IQR)	76.00 (63, 86)	79.00 (68, 86)	< 0.001
Male sex—no. (%)	6725 (53.9)	4526 (55.2)	0.054
Systolic blood pressure—median, mmHg (IQR)	126 (110, 145)	130 (112, 149)	< 0.001
Diastolic blood pressure—median, mmHg (IQR)	69.00 (60, 78)	68 (58, 78)	0.394
Hypertension—no. (%)	4130 (33.1)	3186 (38.9)	< 0.001
Dyslipidemia—no. (%)	2506 (20.1)	1953 (23.8)	< 0.001
Ischemic heart disease—no. (%)	2047 (16.4)	2168 (26.5)	< 0.001
Diabetes—no. (%)	2259 (18.1)	1789 (21.8)	< 0.001
Heart failure—no. (%)	1275 (10.2)	1496 (18.3)	< 0.001
Cancer—no. (%)	2284 (18.3)	1122 (13.7)	< 0.001
Atrial fibrillation—no. (%)	1430 (11.5)	1464 (17.9)	< 0.001
Post cerebrovascular accident—no. (%)	1735 (13.9)	1108 (13.5)	0.459
Chronic renal disease—no. (%)	1476 (11.8)	1269 (15.5)	< 0.001
Chronic obstructive pulmonary disease—no. (%)	1029 (8.2)	867 (10.6)	< 0.001
Peripheral vascular disease—no. (%)	431 (3.5)	398 (4.9)	< 0.001
Gout—no. (%)	175 (1.4)	163 (2.0)	0.001
Anemia—no. (%)	1882 (15.1)	1339 (16.3)	0.015
Liver disease—no. (%)	1342 (10.7)	928 (11.3)	0.202
Dementia—no. (%)	1041 (8.3)	442 (5.4)	< 0.001
Hypothyroidism—no. (%)	742 (5.9)	525 (6.4)	0.182
Valvular disorders—no. (%)	511 (4.1)	516 (6.3)	< 0.001
Pacemaker carriers—no. (%)	344 (2.8)	394 (4.8)	< 0.001
Rheumatoid arthritis—no. (%)	378 (3.0)	293 (3.6)	0.033
Asthma—no. (%)	363 (2.9)	264 (3.2)	0.211
Pulmonary hypertension—no. (%)	171 (1.4)	217 (2.6)	< 0.001
Cardiomyopathy—no. (%)	145 (1.2)	164 (2.0)	< 0.001
Creatinine at admission, mg/dl—median (IQR)	0.99 (0.75, 1.35)	1.12 (0.86, 1.56)	< 0.001
White blood cells count at admission, K/mm^3^—median (IQR)	10.59 (7.43, 14.67)	10.88 (7.95, 14.68)	< 0.001
Hemoglobin at admission, g/dl—median (IQR)	11.47 (10.00, 12.87)	11.60 (10.19, 13.00)	< 0.001
Glucose at admission, mg/dl—median (IQR)	121 (100, 156)	129.00 (106, 171)	< 0.001
Max white blood cells count, K/mm^3^—median (IQR)	12.88 (9.28, 17.40)	13.35 (9.97, 17.77)	< 0.001
Max C-reactive protein—median (IQR)	147.20 (75.63, 227.15)	140.70 (68.47, 224.40)	0.002
Max temperature—median (IQR)	38.00 (37.30, 38.70)	37.90 (37.20, 38.60)	0.010

IQR, interquartile range; SD, standard deviation; mmHg, millimeter of mercury; BPM, beats per minute.

**Table 2:** Characteristics of patients with troponin data at baseline.

	Non-elevated troponin (N = 4988)	Elevated troponin (N = 3207)	P-value
Age, years—median (IQR)	76 (65, 85)	82.00 (73, 88)	< 0.001
Male sex—no. (%)	2708 (54.3)	1818 (56.7)	0.035
Systolic blood pressure, mmHg—median (IQR)	131 (114, 149)	128 (109, 150)	< 0.001
Diastolic blood pressure, mmHg—median (IQR)	70 (60, 79)	68 (58, 79)	< 0.001
Heart rate, BPM—mean (SD)	89.86 (20.72)	91.14 (25.86)	0.021
Hypertension—no. (%)	1766 (35.4)	1420 (44.3)	< 0.001
Dyslipidemia—no. (%)	1100 (22.1)	853 (26.6)	< 0.001
Ischemic heart disease—no. (%)	1022 (20.5)	1146 (35.7)	< 0.001
Diabetes—no. (%)	1007 (20.2)	782 (24.4)	< 0.001
Heart failure—no. (%)	723 (14.5)	773 (24.1)	< 0.001
Cancer—no. (%)	698 (14.0)	424 (13.2)	0.337
Atrial fibrillation—no. (%)	811 (16.3)	644 (20.1)	< 0.001
Post cerebrovascular accident—no. (%)	585 (11.7)	523 (16.3)	< 0.001
Chronic renal disease—no. (%)	586 (11.7)	683 (21.3)	< 0.001
Chronic obstructive pulmonary disease—no. (%)	553 (11.1)	314 (9.8)	0.068
Peripheral vascular disease—no. (%)	193 (3.9)	205 (6.4)	< 0.001
Gout—no. (%)	79 (1.6)	84 (2.6)	< 0.001
Anemia—no. (%)	711 (14.3)	628 (19.6)	< 0.001
Liver disease—no. (%)	494 (9.9)	434 (13.5)	< 0.001
Dementia—no. (%)	228 (4.6)	214 (6.7)	< 0.001
Hypothyroidism—no. (%)	292 (5.9)	233 (7.3)	0.012
Valvular disorders—no. (%)	78 (4.6)	88 (8.5)	< 0.001
Pacemaker carriers—no. (%)	62 (3.6)	80 (7.7)	< 0.001
Rheumatoid arthritis—no. (%)	56 (3.3)	46 (4.4)	0.159
Asthma—no. (%)	58 (3.4)	25 (2.4)	0.167
Pulmonary hypertension—no. (%)	36 (2.1)	37 (3.6)	0.032
Cardiomyopathy—no. (%)	31 (1.8)	33 (3.2)	0.033
Creatinine at admission, mg/dl—median (IQR)	1.03 (0.80, 1.37)	1.33 (0.99, 1.90)	< 0.001
White blood cells count at admission, K/mm^3^—median (IQR)	10.64 (7.69, 14.29)	11.35 (8.41, 15.27)	< 0.001
Hemoglobin at admission, g/dl—median (IQR)	11.80 (10.39, 13.20)	11.30 (9.90, 12.70)	< 0.001
Glucose at admission, mg/dl—median (IQR)	125.00 (104.00, 162.00)	136.00 (110.00, 185.00)	< 0.001

IQR, interquartile range; mmHg, millimeter of mercury; BPM, beats per minute.

### Risk factors for elevated troponin in patients with pneumonia

Adjustment for age, sex, systolic and diastolic blood pressure, creatinine level at admission and comorbidities (heart failure, peripheral vascular disease, chronic obstructive pulmonary disease, cerebrovascular accident and ischemic heart disease), yielded age (odds ratio [OR], 1.19 per 5 years; 95% confidence interval [CI] 1.17–1.22; P-value < 0.001), a prior diagnosis of ischemic heart disease (OR, 1.78; 95% CI 1.58–2.00; P-value < 0.001), increased creatinine level upon admission (OR, 1.65; 95% CI 1.54–1.77; P-value < 0.001), and increased diastolic blood pressure at presentation (OR, 1.10 per 10 mm of mercury [mmHg]; 95% CI 1.05–1.15; P-value < 0.001) as risk factors for elevated troponin. Patients with an increased systolic blood pressure at presentation were at lower risk of an elevated troponin level during their hospitalization (OR, 0.93 per 10 units of mmHg; 95% CI 0.91–0.95; P-value < 0.001), as were also patients with chronic obstructive pulmonary disease (OR, 0.75; 95% CI 0.64–0.89; P-value < 0.01). Sex, background heart failure, peripheral vascular disease and prior stroke were not shown to be statistically significant risk factors for elevated troponin levels. The complete data regarding predictors for elevated troponin in pneumonia are presented in Table 3.

**Table 3:** Predictors for elevated troponin in presence of pneumonia diagnosis.

	Logistic regression models
	Adjusted model OR with 95% CI
Age (per 5 years)	1.19
	(1.17, 1.22)
	p < 0.001
Sex (male)	0.98
	(0.88, 1.10)
	p = 0.79
Systolic pressure (per 10 mmHg)	0.93
	(0.91, 0.95)
	p < 0.001
Diastolic pressure (per 10 mmHg)	1.10
	(1.05, 1.15)
	p < 0.001
First creatinine	1.65
	(1.54, 1.77)
	p < 0.001
Background CHF	1.10
	(0.96, 1.27)
	p = 0.18
Background PVD	1.19
	(0.93, 1.51)
	p = 0.17
Background COPD	0.75
	(0.64, 0.89)
	p < 0.01
Background CVA	1.10
	(0.95, 1.28)
	p = 0.20
Background IHD	1.74
	(1.53, 1.97)
	p < 0.001
Observations	6,748

mmHg, millimeter of mercury; CHF, chronic heart failure; COPD, chronic obstructive pulmonary disease; CVA, cerebrovascular accident; IHD, ischemic heart disease; PVD, peripheral vascular disease.

### Patients' outcome and survival

The median length of stay in the hospital in those with elevated troponin was longer by 2 days than in those with normal troponin (6 days versus 4 days, P-value < 0.001). Patients hospitalized with pneumonia who had elevated troponin levels were more likely to undergo cardiac catheterization during hospitalization than those with non-elevated troponin levels (1.2% versus 0.1%. P-value < 0.001), with similarly low rates of percutaneous coronary intervention (0.2% versus 0.1%. P-value = 0.094).

**Figure 1:**
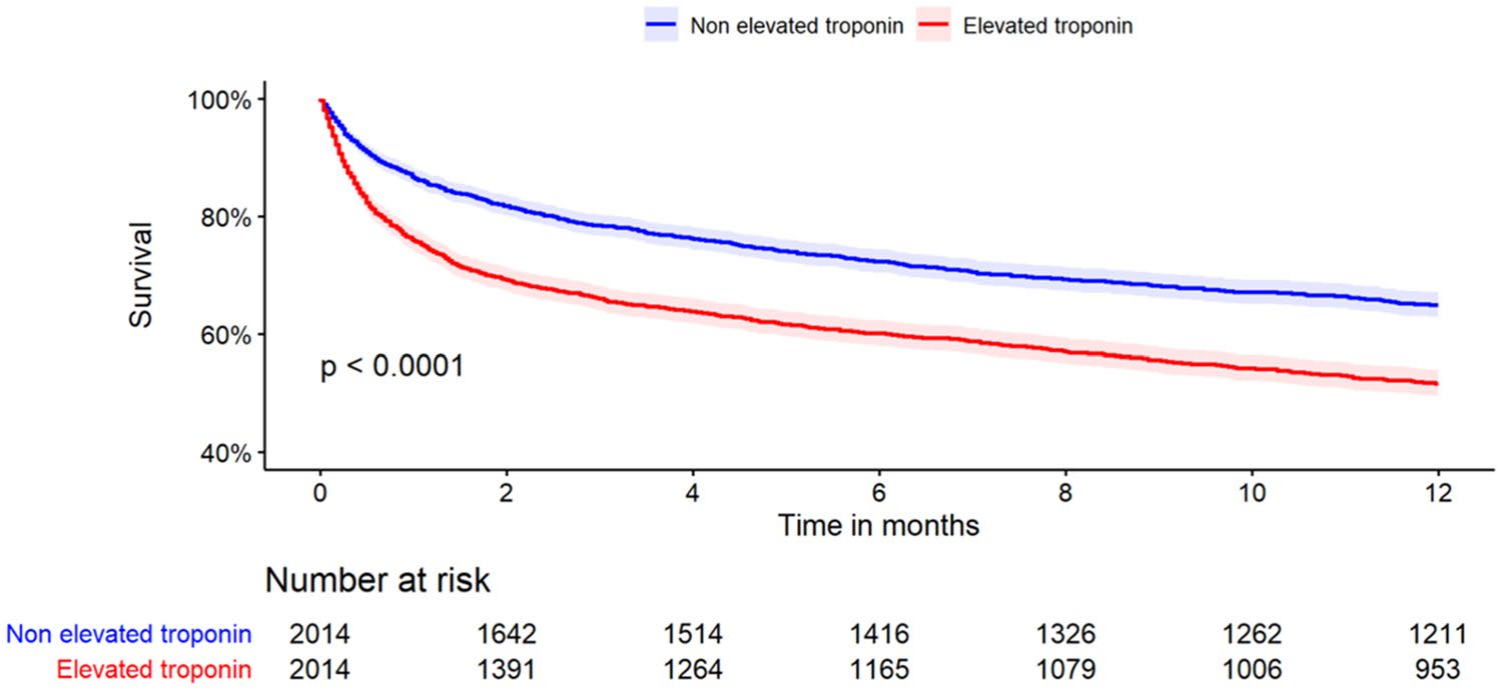
Kaplan–Meier survival curves for elevated and non-elevated troponin in patients hospitalized with pneumonia.

Comparison between patients with elevated troponin and those with normal troponin was made using a propensity score-based matched analysis (area under the curve, 0.73). The matched populations included 4028 patients. Baseline characteristics and mortality of the matched population is shown in Table 4. The in-hospital mortality in those with elevated troponin was almost two folds higher than in those with normal troponin (17.2% versus 8.7%, P-value < 0.001). One-year mortality rate showed a similar trend with a 47.6% mortality rate among those with elevated troponin group as compared to 34.6% in those with normal troponin (P-value < 0.001). A Kaplan–Meier plot demonstrating survival in the propensity score matching is shown in Fig. 1. A Cox regression analysis made on the matched population showed a significantly increased risk of death 1-year after hospitalization (hazard ratio [HR], 1.57; 95% CI 1.42–1.73, P-value < 0.001) (Supplementary 3–4). Similar results were demonstrated when using an adjusted population with significantly increased mortality risk among patients diagnosed with pneumonia and elevated troponin levels as compared to non-elevated troponin levels after 1 year (HR, 1.46; 95% CI 1.32–1.61; P-value < 0.001).

**Table 4:** Characteristics of matched population of patients hospitalized with pneumonia with troponin data at baseline.

	Non-elevated troponin (N = 2014)	Elevated troponin (N = 2014)
Median age, years (IQR)	81.00 (73, 87.75)	82.00 (72, 88)
Male sex—no. (%)	1105 (54.9)	1113 (55.3)
Systolic blood pressure, mmHg—median (IQR)	131.00 (113, 149)	130 (111, 150)
Diastolic blood pressure, mmHg—median (IQR)	68.00 (60, 78)	68.00 (58, 79)
Creatinine at admission, mg/dl—median (IQR)	1.18 (0.90, 1.62)	1.20 (0.91, 1.64)
Hypertension—no. (%)	872 (43.3)	838 (41.6)
Dyslipidemia—no. (%)	525 (26.1)	512 (25.4)
Ischemic heart disease—no. (%)	608 (30.2)	615 (30.5)
Diabetes—no. (%)	471 (23.4)	454 (22.5)
Heart failure—no. (%)	432 (21.4)	410 (20.4)
Cancer—no. (%)	279 (13.9)	280 (13.9)
Atrial fibrillation—no. (%)	404 (20.1)	416 (20.7)
Post cerebrovascular accident—no. (%)	312 (15.5)	311 (15.4)
Chronic renal disease—no. (%)	369 (18.3)	323 (16.0)

IQR, interquartile range; mmHg, millimeter of mercury.

## Discussion

This is a large retrospective cohort study in the largest tertiary care hospital in Israel comparing the prognosis of hospitalized patients with pneumonia in the presence of myocardial injury, as heralded by elevated troponin levels, with patients without elevated troponin levels.

Our study shows that 39.1% of the patients with pneumonia have elevated troponin levels during their hospitalization. Patients with elevated troponin levels were older and had higher rates of underlying ischemic heart disease, cardiovascular risk factors (hypertension, dyslipidemia, chronic renal disease, diabetes, gout, peripheral vascular disease, cerebral vascular disease) and other cardiac diseases (cardiomyopathy, valvular disease, heart failure, atrial arrhythmias) than those with normal troponin levels.

Patients who had elevated troponin were hospitalized longer and were at a higher risk for in-hospital and 1-year mortality than those with normal troponin levels. The data was consistent when the multivariable adjustment model was applied. Several studies have shown that critically ill patients with elevated troponin are at a higher risk of death^12,13^. In accordance with our results, Viasus et al. presented a poor in-hospital prognosis among pneumonia patients with cardiac event on a small investigated cohort^11^. Cangemi et al. further demonstrated a high long term mortality in pneumonia patients with cardiac injury^9^.

We showed that age, prior diagnosis of ischemic heart disease, creatinine level upon admission, and diastolic blood pressure levels are risk factors to develop cardiac injury during pneumonia. Several studies have investigated the risk factors for cardiac injury in the setting of pneumonia. Since the definition of pneumonia and cardiac injury were different between studies, different risk factors were attained. Nevertheless, as was shown in our study, age and prior cardiac disease were important risk factors in all of these studies^10,11^.

Patients with increased systolic blood pressure at presentation were at lower risk of an elevated troponin level. It was previously demonstrated that patients with reduced systolic blood pressure at admission (i.e., < 90 mmHg) had an increased 30-day mortality risk^14,15^.

Chronic obstructive pulmonary disease (COPD) was associated with a lower risk for elevated troponin levels among patients hospitalized with pneumonia. We assume that patients with COPD are hospitalized with less severe pneumonia and are more often tested for troponin levels, as COPD is unlikely to have a protective effect on the myocardium.

Interestingly, there was not a significant increase in the risk for myocardial injury with several cardiovascular risk factors such as peripheral vascular disease and prior stroke.

Previous studies showed that myocardial injury is associated with increased hospital length of stay, multi-organ failure, and an increase in short-term mortality. However, it is not established whether the poor prognosis is derived from the myocardial injury itself, or whether it is a marker of a severe illness. This study shows that myocardial injury itself, as shown by measurement of elevated troponin levels, is a major contributor to poor prognosis among patients hospitalized with a diagnosis of pneumonia. Furthermore, it contributes to lower survival rates both in-hospital and 1-year after hospitalization. This can further direct intensity of care during hospital stay and on a 1-year follow-up after hospital discharge. Furthermore, similar low performance rates of percutaneous coronary intervention among patients with pneumonia who underwent cardiac catheterization during hospitalization were observed with either elevated and non-elevated troponin level, suggesting that cardiac catheterization is not beneficial in those patients during hospital stay.

Clinical evidence of myocardial ischemia (e.g. symptoms or electrocardiogram changes) is likely to be underreported or misclassified in the setting of acute illnesses such as severe pneumonia requiring hospitalization. Thus, this research results may suggest measuring troponin levels in patients hospitalized with pneumonia, even without clinical suspicion of myocardial injury, in order to assess prognosis and adjust clinical care accordingly. Measurement of troponin levels can be guided by the risk factors shown in this study to be associated with greater risk for myocardial injury.

A few limitations exist in this retrospective cohort. First, Myocardial injury as indicated by elevated troponin levels was not differentiated from MI, as there was no analysis of clinical correlation or electrocardiogram changes. Furthermore, the study was conducted in a single medical facility, without unified indications of measuring troponin levels. Therefore, a selection bias may occur as the patients who were measured for troponin levels were selected according to clinical judgment. These patients were older, suffering from more background diseases and their clinical presentation was probably more severe or suggestive of acute cardiac ischemia. To overcome this selection bias, propensity score matching was performed. Nonetheless, it probably presents a problem of generalizing the research conclusion on all patients hospitalized with pneumonia.

Also, troponin levels were not referred as a continuous variable, rather than a binary variable. It cannot be ruled out that troponin maximal level during hospitalization may have an effect on patients’ prognosis, with lower levels being less clinically significant.

Myocardial injury as foreshowed by elevated troponin level has a major impact on prognosis in patients hospitalized with pneumonia. The mortality risk of these patients is higher not only during hospital stay, but also 1-year post hospitalization. Hence, troponin levels may be used as another tool of risk stratification for patients hospitalized with pneumonia. Several major risk factors were found as significant predictors for troponin elevation among these patients. Therefore, troponin measurement is to be considered in all patients hospitalized with a diagnosis of pneumonia. A prospective multicenter study is required to further evaluate prognostic significance of troponin levels in patients hospitalized with pneumonia, and in order to assess the benefits of intensive care and follow-up among these patients.

## Methods

### Trial design and population

The protocols of this study were approved by the Ethical Committee of the Sheba Medical Center, Israel and the study has been conducted according to the principles of the Declaration of Helsinki. Informed consent was waived by the Sheba Medical Center institutional review board committee.

In this retrospective cohort study, data were obtained from the Sheba Medical Center patient registry. We retrieved all consecutive patients who were hospitalized in internal medicine departments in Sheba Medical Center between the years 2008–2019 with a diagnosis of pneumonia. We included patients hospitalized with pneumonia as the primary diagnosis at admission or at discharge. Pneumonia was defined on the basis of diagnosis code 480–488 in the International Classification of Diseases, 9th Revision (ICD-9) or J09-J18 in the International Classification of Diseases, 10th Revision (ICD-10). All patients included were 18 years old or older at the time of admission.

We restricted the analysis to the first event by excluding repeated hospitalizations of the same patient. Since the reference range of normal troponin levels has changed during the study period, an elevated troponin level was defined as any value above the normal reference range according to the hospital laboratory index at the specific period. In 2008–2015 the upper normal value was 0.07 and in 2015–2019 0.059. Therefore, the search was adapted to the normal values according to the time it was measured, rather than an absolute value. Elevated troponin was referred as the highest value from the time of index-hospitalization. Patients with outliers of the troponin highest values (> 100) were excluded.

For each patient, baseline demographic characteristics and clinical information were extracted from the medical records. Clinical data included medical comorbidities, home medications, laboratory tests, duration of hospital stay, vital signs (body temperature, heart rate, systolic blood pressure, diastolic blood pressure), mortality. When death occurred outside the hospital, mortality dates were obtained from the Ministry of internal mortality records.

### Outcomes

We assessed the length of hospital stay, in-hospital mortality and 1-year mortality. We also examined potential predictors of cardiac injury as heralded by elevated troponin level among patients hospitalized with pneumonia who were taken troponin levels during their stay.

### Statistical analysis

Baseline clinical data with normal and non-normal distributed continuous variables and length of stay were compared between groups with and without troponin data, and in-between patients with troponin data (elevated troponin and non-elevated troponin) using the t-test and the Mann–Whitney–Wilcoxon test. Categorial variables were subjected to the Chi-square test. Comparing the two groups of elevated and non-elevated troponin was made using chi-square for categorical variables and with ANOVA or Kruskal–Wallis test as appropriate for normal and non-normal distributed continues variables. Survival analysis comparison was performed using the Kaplan Meier curves followed by the Log Rank test. Investigation of the relationship between survival, study groups, and other explanatory covariates was made using Cox proportional hazard models. To determine whether there is an association between elevated troponin levels among patients hospitalized with pneumonia and mortality, a propensity score-based matched analysis was performed. Propensity score matching was based on a list of variables (Supplementary 1) with a caliper of 0.05. Variables were selected from baseline characteristics (p < 0.05), excluding variables with too many missing values.

Logistic regression was applied to identify variables that are best associated with an elevated Troponin I level following pneumonia diagnosis. Covariates for the multivariable models were pre-specified. P values < 0.05 were considered to be significant. Data analyses were performed using the R Development Core Team, version 3.6.1, Vienna, Austria^16^.

## Supplementary Information

Supplementary Information.
