# Supplementary material for: Risk factors and mortality in patients with pneumonia and elevated troponin levels
Source: Sci Rep. 2020 Dec 10;10:21619. doi: 10.1038/s41598-020-78287-1 (PMC7729902; doi:10.1038/s41598-020-78287-1)
Supplement: Supplementary file 1 — Supplementary Information. [file 41598_2020_78287_MOESM1_ESM.pdf]

Risk Factors and Mortality in Patients with Pneumonia and Elevated Troponin Levels

Orly Efros [1,2*], MD; Shelly Soffer [2], MD; Avshalom Leibowitz [1,2] MD; Alexander Fardman, MD[2,3]; Robert Klempfner [2,3], MD; Eshcar Meisel[1,2], MD PhD; Ehud Grossman [2,4], MD

[1] Internal Medicine D, Sheba Medical Center, Tel-Hashomer Israel

[2] Sackler Faculty of Medicine, Tel-Aviv University, Tel-Aviv, Israel

[3] Cardiac Rehabilitation Institute, Leviev Heart Center, Sheba Medical Center, Tel Hashomer, Israel

[4] Internal medicine wing, Sheba Medical Center, Tel-Hashomer Israel

*Corresponding author: orlyefros@gmail.com

Supplementary 1. Variables used for propensity score matching (1:1)

Age, sex, systolic blood pressure, diastolic blood pressure, hemoglobin level upon admission, creatinine level upon admission, glucose level upon admission, white blood cells count upon admission, days in hospital, pulse rate, background diseases (hypertension, dyslipidemia, ischemic heart disease, diabetes mellitus, renal disease, anemia, atrial arrhythmias, cerebrovascular accident, heart failure, liver disease, dementia, hypothyroidism, valve disorders, peripheral vascular disease, pacemaker, rheumatoid arthritis, asthma, pulmonary hypertension, gout, cardiomyopathy, cancer, chronic obstructive pulmonary disease).

	Non-elevated troponin (n=4988)	Elevated troponin (n=3207)	P-value
ACE Inhibitors - no. (%)	1219 (24.4)	918 (28.6)	<0.001
Antihyperglycemic - no. (%)	91 (1.8)	82 (2.6)	0.030
Selective Beta Blocking Agents - no. (%)	1832 (36.7)	1375 (42.9)	<0.001
HMG-CoA Reductase Inhibitors- no. (%)	2207 (44.2)	1513 (47.2)	0.010

Supplementary 2. Medication of Hospitalized Pneumonia Patients with Troponin Data at Admission
Abbreviations: ACE, Angiotensin-converting enzyme

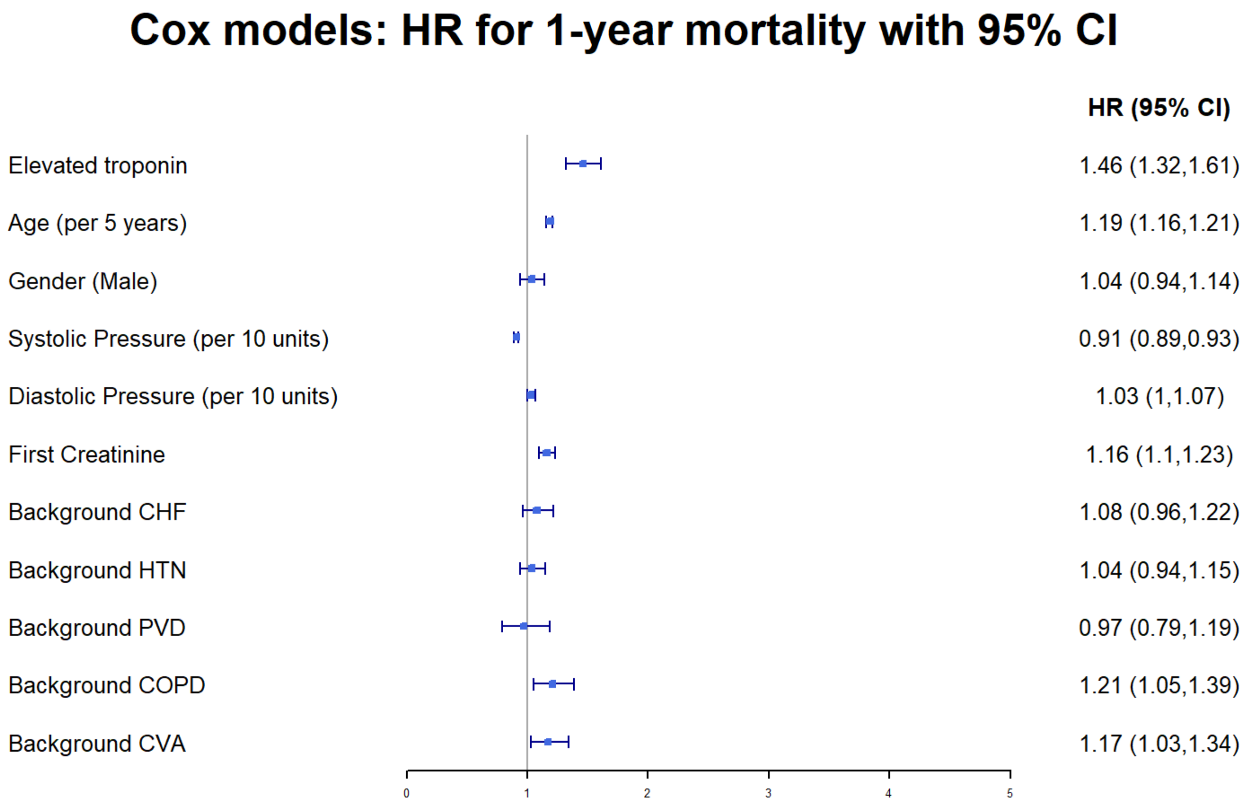

Supplementary 3. Cox Model of Hazard Ratio for 1-Year Mortality of Patients Diagnosed with Pneumonia and Elevated Troponin (Adjusted Model)
Abbreviations: CHF, chronic heart failure; COPD, chronic obstructive pulmonary disease; CVA, cerebrovascular accident; IHD, ischemic heart disease; PVD, peripheral vascular disease.

	Cox Models	
	**—Crude Model—**	**—Adjusted model—**
Elevated troponin	1.85	1.46
	(1.70, 2.01)	(1.32, 1.61)
	p <0.001	p <0.001
Age (per 5 years)		1.19
		(1.16, 1.21)
		p <0.001
Sex (Male)		1.04
		(0.94, 1.14)
		p = 0.46
Systolic Pressure (per 10 units)		0.91
		(0.89, 0.93)
		p <0.001
Diastolic Pressure (per 10 units)		1.03
		(1.00, 1.07)
		p = 0.08
First Creatinine		1.16
		(1.10, 1.23)
		p <0.001
Background CHF		1.08
		(0.96, 1.22)
		p = 0.19
Background HTN		1.04
		(0.94, 1.15)
		p = 0.48
Background PVD		0.97
		(0.79, 1.19)
		p = 0.78
Background COPD		1.21
		(1.05, 1.39)
		p <0.01
Background CVA		1.17[*]
		(1.03, 1.34)
		p = 0.02
Observations	7,318	6,013

Supplementary 4. Cox Model of Hazard Ratio for 1-Year Mortality of Patients Diagnosed with Pneumonia and Elevated Troponin (Adjusted Model)
Abbreviations: CHF, chronic heart failure; COPD, chronic obstructive pulmonary disease; CVA, cerebrovascular accident; IHD, ischemic heart disease; PVD, peripheral vascular disease.
